# Clinical Characteristics of Inpatients With New-Onset Diabetes Mellitus in Eastern China: Based on Novel Clustering Analysis

**DOI:** 10.3389/fendo.2022.927661

**Published:** 2022-07-27

**Authors:** Xueke Song, Yingqi Lv, Nan Huang, Jinfang Sun, Ting Yang, Xiaoyuan Wang, Jianan Zhang, Ziwei Zhou, Huihua Gao, Jie Li, Wei Zhang, Han Yin, Qiong Wei, Kun Wang, Ling Li

**Affiliations:** ^1^Department of Endocrinology, Zhongda Hospital, Southeast University, Nanjing, China; ^2^Institute of Glucose and Lipid Metabolism, Southeast University, Nanjing, China; ^3^MoE Key Laboratory of Environmental Medicine Engineering, School of Public Health, Southeast University, Nanjing, China; ^4^Department of Internal Medicine, Xigang Community Health Service Center, Nanjing, China; ^5^Department of Endocrinology, Nanjing Central Hospital, Nanjing, China; ^6^Department of Endocrinology, Second People’s Hospital of Wuhu, Wuhu, China; ^7^Department of Endocrinology, Nanjing Jiangning Hospital, Nanjing, China

**Keywords:** clustering analysis, diabetes, precise treatments, Eastern China, inpatients

## Abstract

**Introduction:**

This study aimed to explore the novel classification of inpatients with new-onset diabetes in Eastern China by the cluster-based classification method and compare the clinical characteristics among the different subgroups.

**Methods:**

A total of 1017 Inpatients with new-onset diabetes of five hospitals in Eastern China were included in the study. Clustering analysis was used to cluster the data into five subgroups according to six basic variables. The differences in clinical characteristics, treatments, and the prevalence of diabetes-related diseases among the five subgroups were analyzed by multiple groups comparisons and pairwise comparisons. The risk of diabetes-related diseases in the five subgroups was compared by calculating odd ratio (OR). *P* value < 0.05 was considered significant.

**Results:**

Five subgroups were obtained by clustering analysis with the highest proportion of patients with severe insulin-deficient diabetes (SIDD) 451 (44.35%), followed by patients with mild age-related diabetes (MARD) 236 (23.21%), patients with mild obesity-related diabetes (MOD) 207 (20.35%), patients with severe insulin-resistant diabetes (SIRD) 81 (7.96%), and patients with severe autoimmune diabetes (SAID) 42 (4.13%). Five subtypes had their own unique characteristics and treatments. The prevalence and risk of diabetes-related complications and comorbidities were also significantly different among the five subtypes. Diabetic kidney disease (DKD) was the most common in SIRD group. Patients in SIDD, SIRD, and MARD groups were more likely to develop cardiovascular disease (CVD) and/or stroke, diabetic peripheral vascular disease (DPVD), and diabetic distal symmetric polyneuropathy (DSPN). The prevalence and risk of metabolic syndrome (MS) were the highest in MOD and SIRD groups. Patients in SAID group had the highest prevalence and risk of diabetic ketoacidosis (DKA). Patients with MOD were more likely to develop non-alcoholic fatty liver disease (NAFLD).

**Conclusions:**

The inpatients with new-onset diabetes in Eastern China had the unique clustering distribution. The clinical characteristics, treatments, and diabetes-related complications and comorbidities of the five subgroups were different, which may provide the basis for precise treatments of diabetes.

## 1 Introduction

With the global economic development and population aging trend, the number of patients with diabetes mellitus has risen sharply worldwide (1). The disease has become a critical health concern all over the world owing to its high prevalence and related disability and mortality (2). However, existing treatment strategies have been unable to prevent the progression of the disease and the development of its related complications and comorbidities. In addition to gestational diabetes and other special types of diabetes, diabetes was conventionally classified into type 1 diabetes mellitus and type 2 diabetes mellitus, but type 2 diabetes mellitus were highly heterogeneous (3). The clinical manifestations, response to treatments, metabolic control, occurrence and development of complications and comorbidities, severity, and prognosis of type 2 diabetes mellitus might vary widely (4). The current classification system was far from meeting the needs of clinicians and patients for precise treatments of diabetes (5, 6). Therefore, the accurate classification of diabetes contributes to the individualized development of clinical treatment strategies and plays a crucial role in the management of chronic diseases. Findings of a Swedish cohort study challenged the current paradigm of classifying patients with diabetes (7). They used clustering analysis that identified five exclusive diabetes subgroups as severe autoimmune diabetes (SAID), severe insulin-deficient diabetes (SIDD), severe insulin-resistant diabetes(SIRD), mild obesity-related diabetes (MOD), and mild age-related diabetes (MARD) according to six variables including age at onset of diabetes, body mass index (BMI), glycated hemoglobin A1c (HbA1c), homoeostatic model assessment 2 estimates of β-cell function index (HOMA2-β), homoeostatic model assessment 2 estimates of insulin resistance index (HOMA2-IR), and presence or absence of glutamic acid decarboxylase antibody (GADA). The study also found that these five subgroups had different disease progression and the risk of diabetes-related complications (7). At present, the stability and applicability of this new cluster-based classification have been validated in populations of multiple regions, ethnicities, and disease backgrounds (8–12). This new cluster-based classification provided a new clinical idea that helped to bring personalized medicine to the forefront of treatments, and might reduce the risk of diabetes-related complications and comorbidities.

This multicenter study was a three-year cross-sectional observational study aimed at (1) exploring the new classification of hospitalized patients with diabetes in Eastern China by the novel cluster-based classification method, (2) comparing the differences in clinical characteristics, treatments, and diabetes-related complications and comorbidities among the five subgroups.

## 2 Methods

### 2.1. Study Population

Medical service institutions are divided into three levels according to their scales and functions in China. First level medical service institutions are community-centered primary health care institutions. Second level medical service institutions are regional hospitals centered on autonomous cities or districts to provide comprehensive medical services for multiple communities. Third level medical service institutions are large-scale hospitals that provide high-level specialized medical services for several regions and carry out higher education and scientific research tasks. This study enrolled hospitalized patients with new-onset diabetes in the Department of Endocrinology at Zhongda Hospital Affiliated to Southeast University (third level medical service institution), Affiliated Jiangning Hospital of Nanjing Medical University (third level medical service institution), The Second People’s Hospital of Wuhu (third level medical service institution), Nanjing Central Hospital (second level medical service institution), and Xigang Community Health Service Center (first level medical service institution) from July 2018 to July 2021. All patients included in the study met the following inclusion criteria: (1) patients were first diagnosed with diabetes (duration of diabetes ≤ 2 years) based on the 1999 criteria of the WHO (13), (2) Data of fasting plasma glucose (FPG) and postprandial blood glucose (PBG) at diagnosis was available, (3) Without any antihyperglycemic drugs before hospitalization. Exclusion criteria: (1) active infection, (2) serious other systemic diseases, (3) receiving glucocorticoid, (4) diagnosed as gestational diabetes and other special types of diabetes, (5) incomplete relevant clinical data. All patients were eligible for this study signed informed consent documents. The study was approved by the ethics committees of the hospitals.

### 2.2. Research Contents

Demographic information including age, sex, emaciation, symptoms of polydipsia, polyuria, and polyphagia (3P), smoking status, alcohol, and diabetic family history (DFH) were inquired and recorded by professional resident physicians on the day of hospitalization. After resting quietly for at least five minutes, systolic blood pressure (SBP) and diastolic blood pressure (DBP) were measured and recorded with the standard electronic sphygmomanometer by primary nurses. In the morning of the next day (at least eight hours after fasting), the height and weight of participants with thin clothes and trousers were measured and recorded by primary nurses.

In the morning of the next day (at least eight hours after fasting), peripheral venous blood samples of 5-10 ml were collected of all subjects to test related laboratory indexes. HbA1c was measured by high performance liquid chromatography with HbA1c analyzers (BIO-RAD D-10). The alanine aminotransferase (ALT), aspartate aminotransferase (AST), alkaline phosphatase (ALP), γ-glutamyl transpeptidase (GGT) were measured by rate assay with automatic biochemical analyzers (BECKMAN COULTER AU5821/BECKMAN COULTER AU5421/HITACHI 7180). The glycosylated albumin (GA), triglyceride (TG), total cholesterol (TC), high density lipoprotein cholesterol (HDL-C), low density lipoprotein choleste (LDL-C), serum uric acid (SUA) were measured by endpoint method with automatic biochemical analyzers (BECKMAN COULTER AU5821/BECKMAN COULTER AU5421/HITACHI 7180). The serum creatinine (SCr) was detected by picric acid with automatic biochemical analyzers (BECKMAN COULTER AU5821/BECKMAN COULTER AU5421/HITACHI 7180). The platelet (PLT) was detected by light scattering technique with automatic blood cell analyzers (BECKMAN COULTER DXH600/800). Diabetes-related auto-antibodies including GADA, zinc transporter 8 antibody (ZnT8A), protein tyrosine phosphatase 2 antibody (IA-2A), islet cell antibody-40KD (ICA-40KD), islet cell antibody-120KD (ICA-120KD), islet cell antibody-64KD (ICA-64KD), and insulin autoantibody-5.8KD (IAA-5.8KD) were measured by enzymelinked immunosorbent assay with Western blotting kit (BLOT). All participants underwent oral glucose tolerance test (OGTT) to measure fasting C-peptide (FC-p), 30-minute postprandial C-peptide (30-min PC-p), 60-minute postprandial C-peptide (60-min PC-p), 120-minute postprandial C-peptide (120-min PC-p), and 180-minute postprandial C-peptide (180-min PC-p) by electrochemiluminescence immunoassay with chemiluminescent analyzers (ROCHE COBASE 601) and FPG and PBG by endpoint method with automatic biochemical analyzers (BECKMAN COULTER AU5821/BECKMAN COULTER AU5421/HITACHI 7180). The above laboratory variables were analyzed in the centers of Clinical Laboratory of all participating hospitals according to the standard methods. All centers of Clinical Laboratory implements internal and external quality control procedures directed by a Chinese Quality Control Laboratory.

After completing the above laboratory tests, professional physicians formulated the baseline antihyperglycemic treatments for the first time based on clinical experience. Data was also collected on antihyperglycemic drugs of baseline treatments in each participant, including metformin, sulfonylureas (SU), glinides, thiazolidinedione (TZD), insulin, GLP-1 receptor agonists, SGLT-2 inhibitors, DPP-4 inhibitors, and α-glucosidase inhibitors. Data was collected by uniformly trained professional resident physicians at each center.

HOMA2-β and HOMA2-IR were calculated by FPG and FC-p using the following formulas: HOMA2-β = 0.27 × FC-p [pmol/L]/(FPG [mmol/L]—3.5) and HOMA2-IR = 1.5 + (FPG [mmol/L] × FC-p [pmol/L]/2800) (12, 14). Body mass index (BMI) was calculated using the following formula: BMI = body weight (kg)/body height (m^2^) (15). Two highly recognized noninvasive liver fibrosis indexes were calculated from routine laboratory variables as per the formulas given below: 1. aspartate aminotransferase to platelet ratio index (APRI) = [AST (U/L)/normal upper limit reference value × 100]/[PLT (× 10^9^/L)] (16). 2. γ-glutamyl transpeptidase to platelet ratio (GPR) = [GGT (U/L)]/[PLT (× 10^9^/L)] (17). The estimated glomerular filtration rate (eGFR) level was calculated using the modification of diet in renal disease (MDRD) equation for Chinese patients. The following formula was used: eGFR (ml/min/1.73 m^2^) = 175 × SCr (mg/dl)^−1.234^ × old (years)^−0.179^ × (0.79 if female) (18).

### 2.3. Definitions of Diabetes-Related Complications and Comorbidities

All participants were screened for diabetes-related complications. Diabetic retinopathy (DR) was diagnosed by professional ophthalmologists based on retina fundus photographs taken by non-mydriatic retina fundus cameras (19). The diagnostic criteria of diabetic kidney disease (DKD) were glomerular filtration rate (GFR) < 60 ml/min/1.73 m^2^ and/or urinary albumin to creatinine ratio (UACR) ≥ 30 mg/g for more than three months, excluding chronic kidney diseases due to other causes (20, 21). Ankle brachial index (ABI) and transcutaneous oxygen pressure (TcPO2) were measured by arteriosclerosis diagnostic instrument and transcutaneous oxygen pressure detector respectively. ABI < 0.9 and/or TcPO2 < 40 mmHg suggested diabetic peripheral vascular disease (DPVD) (22, 23). Diabetic distal symmetric polyneuropathy (DSPN) was diagnosed by measuring ankle reflex, acupuncture pain perception, vibration perception, pressure perception, and temperature perception according to the Chinese guideline for the prevention and treatment of type 2 diabetes mellitus (2017 edition) (24). Diabetic ketoacidosis (DKA) was characterized by hyperglycemia (blood glucose > 13.9mmol/L [250mg/dL]), hyperketonemia (serum ketone body ≥3 mmol/L), and metabolic acidosis. Specific diagnostic criteria were referred to the guidelines for diagnosis and therapy of hyperglycemic crisis in China (25). Diabetes-related comorbidities collected in this study included cardiovascular diseases (CVD) and/or stroke, metabolic syndrome (MS), and non-alcoholic fatty liver disease (NAFLD). The histories of CVD and/or stroke were determined by inquiring medical histories and collecting previous medical records. The diagnostic criteria of MS referred to the recommendations of Chinese Diabetes Society (26). NAFLD was diagnosed by experienced sonographers using the high-resolution ultrasound imaging system, after excluding excessive alcohol and previous history of related liver diseases (27).

### 2.4. Statistical Analyses

Statistical analyses were conducted using SPSS software version 26 (IBM NY). We used two step clustering analysis to cluster the data into five subgroups according to six variables, including age at diagnosis, BMI, HbA1c, HOMA2-β, HOMA2-IR, and presence or absence of diabetes-related auto-antibodies. Before this, the five numerical variables were normalized. These five continuous variables of clustering analysis were used as the main outcome indicators to estimate the sample size of comparisons among multiple groups using PASS software version 15 (REACHSOFT BEI JING). Data were presented as frequencies (percentages) for count data, means ± standard deviations for normally distributed continuous variables, and medians (interquartile ranges) for nonnormally distributed continuous variables. For normally distributed continuous variables, the analysis of variance (ANOVA) was performed to assess significant differences among multiple groups. Least significant difference (LSD) or Tamhane T2 test was used for pairwise comparisons based on the test for homogeneity of variance. If the continuous variables were nonnormally distributed, the Kruskal-Wallis test was used for comparisons among multiple groups and the Nemenyi test was used for pairwise comparisons. The chi-squared test and bonferroni correction method were performed to assess significant differences in multiple groups and pairwise groups for the count data, respectively. Logistic regression analysis was used to calculate odd ratio (OR) and 95% confidence interval (CI) of diabetes-related complications and comorbidities. *P* value < 0.05 was considered significant.

## 3 Results

### 3.1. Clustering Analysis

**Figure 1:**
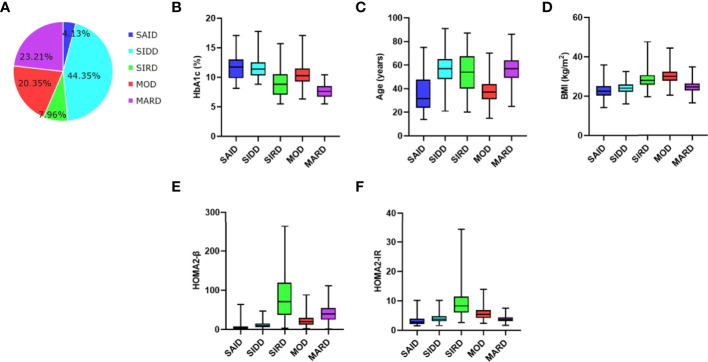
Distribution and clustering characteristics of patients **(A)** Distribution of patients (n, 1017) according to the clustering analysis. Distributions of **(B)** HbA1c, **(C)** age at diagnosis, **(D)** BMI, **(E)** HOMA2‐β, and **(F)** HOMA2‐IR in patients for each cluster of the study. SAID, severe autoimmune diabetes. SIDD, severe insulin-deficient diabetes. SIRD, severe insulin-resistant diabetes. MOD, mild obesity-related diabetes. MARD, mild age-related diabetes. BMI, body mass index. HOMA2-β, homeostasis model assessment 2 estimates of β cell function index. HOMA2-IR, homeostasis model assessment 2 estimates of insulin resistance index.

A total of 1017 inpatients with new-onset diabetes were eligible for this study. According to the clustering analysis, the participants were divided into five subgroups, including 42 (4.13%) patients with SAID, 451 (44.35%) patients with SIDD, 81 (7.96%) patients with SIRD, 207 (20.35%) patients with MOD, and 236 (23.21%) patients with MARD (**Figure 1**). The sample size estimates of comparisons among multiple groups showed that sample sizes of 7, 78, 14, 35, and 40 are obtained from the five groups whose means are to be compared. The total sample of 174 subjects achieves 91% power to detect differences among the means versus the alternative of equal means using an F test with a 0.05 significance level. It can be seen that the sample size of our study is sufficient.

### 3.2. Clinical Characteristics of Different Subgroups

Clustering analysis with six variables revealed that the five subtypes had their own unique characteristics (**Figure 1**). SAID was equivalent to traditional type 1 diabetes mellitus and was characterized by poor metabolic control in blood glucose (the highest HbA1c), early-onset disease, relatively low BMI, overt insulin deficiency (the lowest HOMA2-β), no insulin resistance, and positive diabetes-related auto-antibodies. In the SAID group, there were 24 patients with only one kind of diabetes-related auto-antibodies for GADA, accounting for 57.14%, followed by 10 patients with only one kind of diabetes-related auto-antibodies for ICA-120, accounting for 23.81%, and a few patients with two or three different antibodies at the same time (**Supplementary Table 1**). SIDD was diabetes-related auto-antibodies negative and late-onset disease but otherwise similar to SAID. The characteristics of SIRD were severe insulin resistance (the highest HOMA2-IR), more insulin secretion (the highest HOMA2-β), relatively high BMI, and late-onset age. MOD was characterized by the highest BMI, mild insulin resistance, and early-onset disease. Patients with MARD were diagnosed at the latest age and they were only modest metabolic derangement in blood glucose (the lowest HbA1c) with better islet β-cell function.

The baseline characteristics of different subgroups in addition to the clustering variables were shown in **Table 1**. Among the five subgroups, no significant differences were observed in terms of sex, smoking status, and alcohol (*P* > 0.05). GA, FPG, and PBG were the highest in SAID and SIDD groups (*P* < 0.05 vs the other three groups), while the lowest in MARD group (*P* < 0.05 vs the other four groups). Patients with SAID were more likely to have symptoms of polydipsia, polyuria, polyphagia, and emaciation. Patients with MOD had the strongest genetic susceptibility of diabetes. Patients with SIRD and MOD seemed to have a higher lipid profile (TG), UA, liver function indexes (ALT, AST, and GGT), and recognized markers of noninvasive liver fibrosis (APRI and GPR) compared with those allocated to other clusters. Patients with SIRD and MARD had poor renal function (the lowest eGFR and the highest SCr).

**Table 1:** Baseline characteristics of patients in the five subgroups.

	Total population (n = 1017)	Cluster 1 SAID (n = 42)	Cluster 2 SIDD (n = 451)	Cluster3 SIRD (n = 81)	Cluster 4 MOD (n = 207)	Cluster 5 MARD (n = 236)	*P* value
Female	305 (30%)	16 (38.1%)	136 (30.2%)	25 (30.9%)	51 (24.6%)	77 (32.6%)	0.292
Male	712 (70%)	26 (61.9%)	315 (69.8%)	56 (69.1%)	156 (75.4%)	159 (67.4%)	0.292
SBP (mmHg)	135.31 ± 18.72	125.14 ± 15.78^c^	133.09 ± 18.49^b^	139.41 ± 18.54^a^	138.18 ± 18.29^a^	137.42 ± 19^a^	< 0.001
DBP (mmHg)	82.79 ± 12.64	76.67 ± 11.55^c^	81.45 ± 11.58^b^	85.69 ± 13.28^a^	86.86 ± 13.03^a^	81.86 ± 13.16^b^	< 0.001
Emaciation	438 (43.1%)	31 (73.8%)^a^	251 (55.7%)^b^	21 (25.9%)^d^	86 (41.5%)^c^	49 (20.8%)^d^	< 0.001
3P	625 (61.5%)	39 (92.9%)^a^	317 (70.3%)^b^	38 (46.9%)^c^	135 (65.2%)^b^	96 (40.7%)^c^	< 0.001
Smoking status	388 (38.2%)	12 (28.6%)	176 (39%)	26 (32.1%)	81 (39.1%)	93 (39.4%)	0.512
Alcohol	239 (23.5%)	5 (11.9%)	100 (22.2%)	16 (19.8%)	52 (25.1%)	66 (28%)	0.129
DFH	288 (28.3%)	8 (19%)^b,c^	134 (29.7%)^b^	21 (25.9%)^b,c^	81 (39.1%)^a^	44 (18.6%)^c^	< 0.001
GA (%)	27.27 ± 10.23	33.52 ± 9.77^a^	33.27 ± 9.24^a^	23.05 ± 10.3^b^	25.53 ± 6.65^b^	17.69 ± 4.57^c^	< 0.001
FPG (mmol/L)	13.1 (6.81)	17.4 (9.63)^a^	15.2 (5.2)^a^	11.31 (7.97)^c^	14.2 (6.03)^b^	8.54 (2.6)^d^	< 0.001
PBG (mmol/L)	19.33 (7.85)	24.8 (10.15)^a^	22 (7)^a^	17 (8.98)^c^	20 (6.6)^b^	14.95 (4.3)^d^	< 0.001
FPG (md/dL)	235.8 (122.68)	313.1 (173.34)^a^	273.6 (93.6)^a^	203.58 (143.46)^c^	255.6 (108.54)^b^	153.63 (46.86)^d^	< 0.001
PBG (md/dL)	347.94 (141.3)	446.4 (182.7)^a^	396 (126)^a^	306 (161.55)^c^	360 (118.8)^b^	269.1 (77.4)^d^	< 0.001
ALT (U/L)	26 (25)	20 (25.5)^b^	21 (16)^b^	34 (38)^a^	42 (46)^a^	25 (20.75)^b^	< 0.001
AST (U/L)	21 (14)	19.5 (11.25)^c^	20 (10)^c^	26 (15.5)^a,b^	28 (29)^a^	22 (11)^b^	< 0.001
ALP (U/L)	85 (34)	87.5 (49.75)^a^	89 (33)^a^	85 (32.5)^a,b^	87 (32)^a^	76 (36)^b^	< 0.001
GGT (U/L)	35 (34)	22.5 (23.25)^c^	30 (23)^c^	45 (34.5)^a,b^	51 (46)^a^	37 (40.5)^b^	< 0.001
TG (mmol/L)	1.58 (1.5)	1.05 (0.99)^c^	1.43 (1.1)^c^	1.73 (1.85)^b^	2.55 (3.2)^a^	1.5 (1.35)^b,c^	< 0.001
TC (mmol/L)	4.86 (1.45)	4.72 (1.92)^a,b^	4.91 (1.52)^a,b^	4.9 (1.29)^a,b^	5.16 (1.54)^a^	4.53 (1.18)^b^	< 0.001
LDL-C (mmol/L)	2.91 ± 0.87	2.89 ± 1.06^a,b^	2.93 ± 0.89^a,b^	2.92 ± 0.93^a,b^	3.08 ± 0.83^a^	2.75 ± 0.79^b^	0.004
HDL-C (mmol/L)	1.2 (0.33)	1.32 (0.65)^a^	1.21 (0.32)^a,b^	1.16 (0.32)^a,b^	1.15 (0.31)^b^	1.22 (0.34)^a,b^	0.001
SCr (μmol/L)	62 (22)	57 (22.5)^c^	59 (22)^c^	70 (23.5)^a^	60 (20)^b,c^	64 (21.5)^a,b^	< 0.001
eGFR (ml/min/ 1.73m^2^)	133.15 ± 41.48	149.33 ± 54.54^a^	135.68 ± 39.74^a^	110.08 ± 37.46^b^	145.23 ± 40.97^a^	122.73 ± 38.28^b^	< 0.001
SUA (μmol/L)	329.05 ± 114.15	311.38 ± 144.35^b,c^	292.2 ± 90.08^c^	382.22 ± 141.16^a^	395.1 ± 131.68^a^	326.42 ± 87.67^b^	< 0.001
APRI	0.26 (0.2)	0.24 (0.16)^b,c^	0.23 (0.16)^c^	0.3 (0.24)^a,b^	0.33 (0.3)^a^	0.26 (0.19)^b,c^	< 0.001
GPR	0.17 (0.16)	0.11 (0.11)^d^	0.15 (0.12)^c,d^	0.21 (0.18)^a,b^	0.23 (0.24)^a^	0.18 (0.19)^b,c^	< 0.001

SAID, severe autoimmune diabetes; SIDD, severe insulin-deficient diabetes; SIRD, severe insulin-resistant diabetes; MOD, mild obesity-related diabetes; MARD, mild age-related diabetes; SBP, systolic blood pressure; DBP, diastolic blood pressure; 3P, symptoms of polydipsia, polyuria, and polyphagia; DFH, diabetic family history; GA, glycosylated albumin; FPG, fasting plasma glucose. PBG, postprandial blood glucose. ALT, alanine aminotransferase; AST, aspartate aminotransferase; ALP, alkaline phosphatase. GGT, γ-glutamyl transpeptidase. TG, triglyceride; TC, total cholesterol; LDL-C, low density lipoprotein choleste; HDL-C, high density lipoprotein cholesterol; SCr, serum creatinine; eGFR, estimated glomerular filtration rate; SUA, serum uric acid; APRI, aspartate aminotransferase to platelet ratio index; GPR, γ-glutamyl transpeptidase to platelet ratio. The variables marked with the same letters indicated no significant differences of pairwise comparisons. The variables marked with different letters were used to indicate pairwise comparisons with significant differences. P value < 0.05 was considered significant.

**Figure 2:**
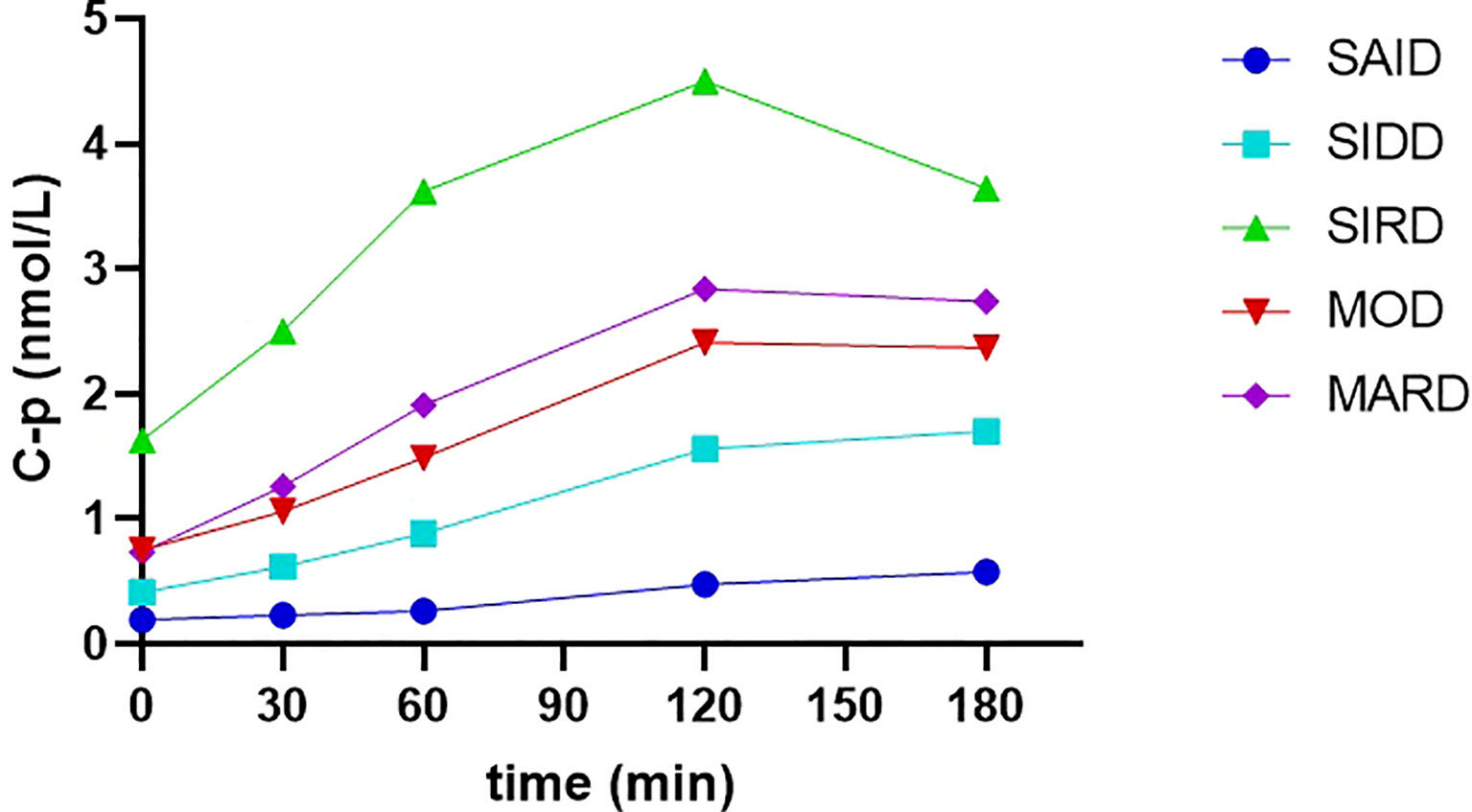
The releasing curves of C-p levels during the OGTT of the five subgroups C-p, C-peptide. SAID, severe autoimmune diabetes. SIDD, severe insulin-deficient diabetes. SIRD, severe insulin-resistant diabetes. MOD, mild obesity-related diabetes. MARD, mild age-related diabetes. C-p was presented as median.

Changes in C-p releasing levels during the OGTT of the five subgroups were shown in **Figure 2**. The total AUC (area under the curve) of 0-180min C-p was the highest in the SIRD group (13.19 [8.27]), followed by MARD, MOD, SIDD, and SAID groups (7.91 [3.88], 6.66[3.26], 4.21[2.54], 1.45[1.82], respectively). It showed significant statistical differences between any two groups in pairwise comparisons (*P* < 0.05).

### 3.3. Differences in Diabetes-Related Complications and Comorbidities Among the Five Subgroups

**Figure 3:**
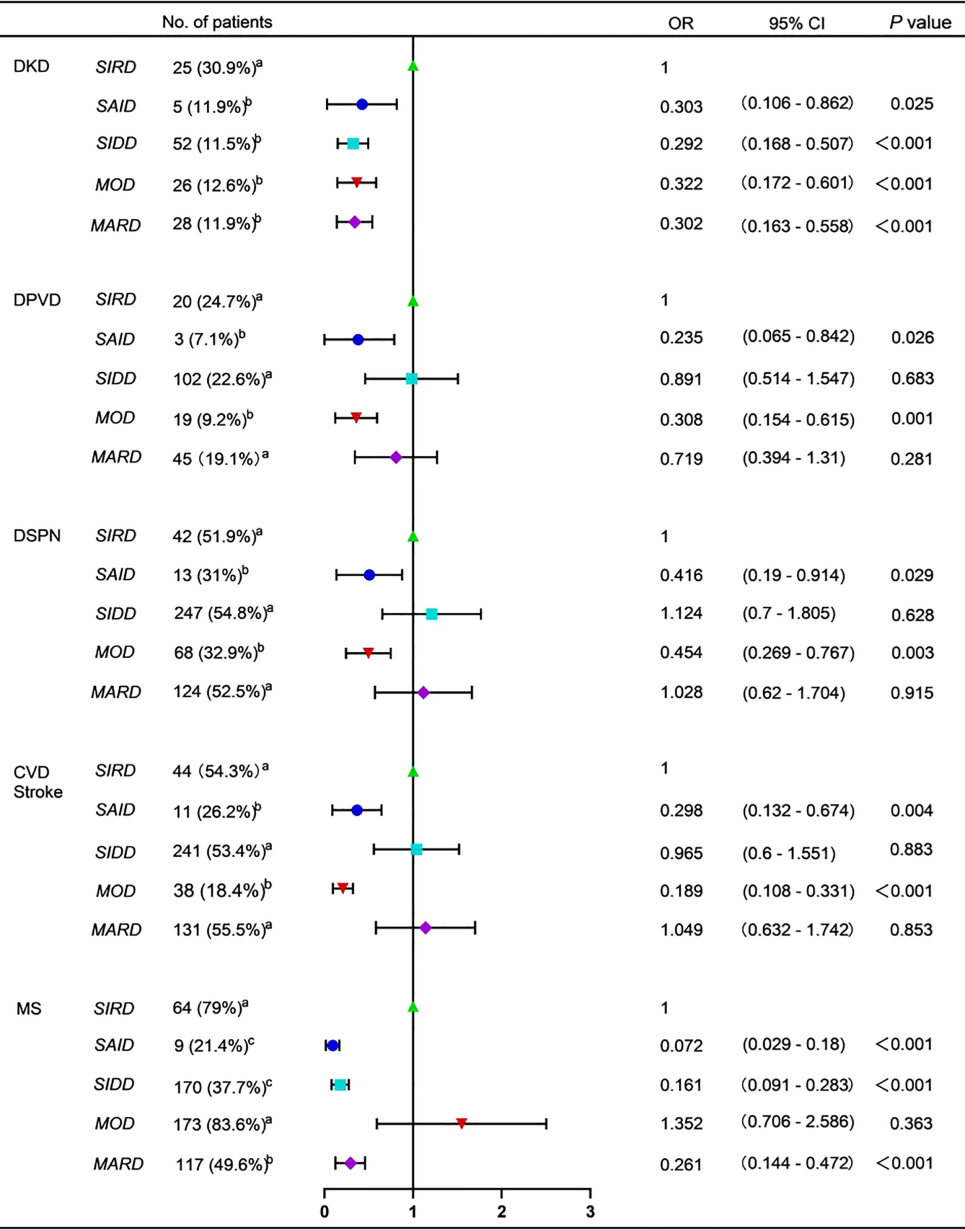
Forest plot for diabetes-related complications and comorbidities DKD, diabetic kidney disease. DPVD, diabetic peripheral vascular disease. DSPN, diabetic distal symmetric polyneuropathy. CVD, cardiovascular diseases. MS, metabolic syndrome. SAID, severe autoimmune diabetes. SIDD, severe insulin-deficient diabetes. SIRD, severe insulin-resistant diabetes. MOD, mild obesity-related diabetes. MARD, mild age-related diabetes. No. of patients with diabetes-related complications and comorbidities were presented as frequencies (percentages). The variables marked with the same letters indicated no significant differences in pairwise comparisons. The variables marked with different letters were used to indicate pairwise comparisons with significant differences. OR, odd ratio. 95% CI, 95% confidence interval. The SIRD group was decided as the control group. *P* value < 0.05 was considered significant.

DKD was the most common in SIRD group (**Figure 3**). The risk of DKD was also the highest in the SIRD group, but no significant difference was seen among the five subgroups in HOMA2-IR-adjusted risk (**Figure 3** and **Supplementary Table 2**). Patients in SIDD, SIRD, and MARD groups were more likely to develop CVD and/or stroke, DPVD, and DSPN (**Figure 3**). However, there was no significant difference in the risk of CVD and/or stroke, DPVD, and DSPN among the five subgroups in the adjusted model with age (**Supplementary Table 2**). The prevalence and risk of MS were the highest in MOD and SIRD groups, but the risk was no longer significantly higher in them after adjusting for HOMA2-IR and BMI (**Figure 3** and **Supplementary Table 2**). There were no significant differences in the prevalence and risk of DR among the five subgroups (*P* > 0.05; **Supplementary Table 3**). Patients in SAID group had the highest prevalence of DKA (16 [38.1%], *P* < 0.05 vs the other four groups; **Supplementary Table 3**), but the risk was no longer significantly higher in them after adjusting for HOMA2-β and HbA1c (**Supplementary Table 3**). NAFLD was the most common in MOD group (190 [91.8%], *P*< 0.05 vs the other four groups; **Supplementary Table 3**). The risk of NAFLD was also the highest in MOD group even after adjusting for BMI and HOMA2-IR (**Supplementary Table 3**).

### 3.4. Differences in Antihyperglycemic Drugs Among the Five Subgroups

**Figure 4:**
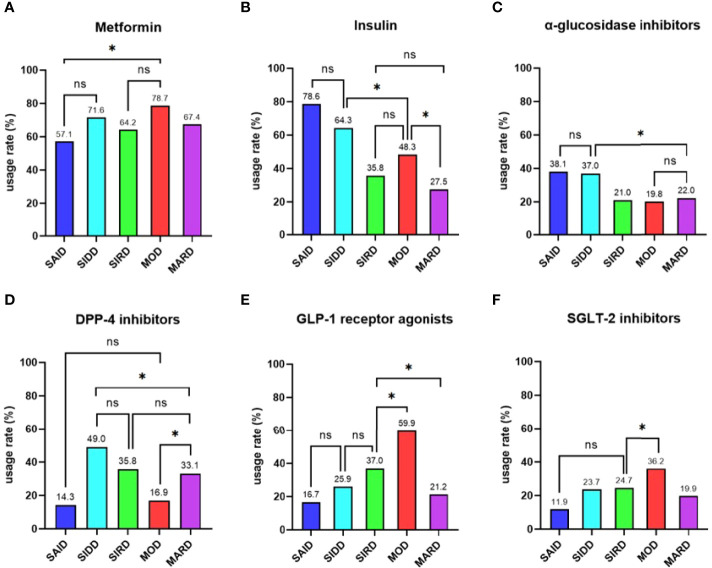
Comparisons of antihyperglycemic drugs among the five subgroups Differences in the usage rates of **(A)** metformin, **(B)** insulin, **(C)** α-glucosidase inhibitors, **(D)** DPP-4 inhibitors **(E)** GLP-1 receptor agonists, and **(F)** SGLT-2 inhibitors among the five subgroups. SAID = severe autoimmune diabetes. SIDD = severe insulin-deficient diabetes. SIRD = severe insulin-resistant diabetes. MOD = mild obesity-related diabetes. MARD = mild age-related diabetes. * indicated pairwise comparisons with significant differences. ns indicated pairwise comparisons without significant differences. P value < 0.05 was considered significant.

Metformin was the most widely used of the nine types of antihyperglycemic drugs in all participants, followed by insulin (721 [70.9%] and 517 [50.8%], respectively). The usage rates of SU, glinides, and TZD were low and there were no significant differences among five subgroups (*P* > 0.05). MOD group had the highest proportion of GLP-1 receptor agonists and SGLT-2 inhibitors (*P* < 0.05 vs the other four groups; **Figure 4**). The utilization rate of DPP-4 inhibitors in SIDD, SIRD and MARD groups was significantly higher than that in SAID and MOD groups (P < 0.05; **Figure 4**). Insulin and α-glucosidase inhibitors were the most common in SAID and SIDD groups (*P* < 0.05 vs the other three groups; **Figure 4**).

## 4 Discussion

The novel classification method with a data-driven clustering analysis of six variables in patients with new-onset diabetes was first used in the Nordic population, and its stability was validated in Chinese, US, German, and Japanese populations (7–11). Recently, a study found that this cluster‐based classification could also be applied to hospitalized adult patients with new-onset diabetes in Beijing, China (12). As far as we know, this is the first study to implement this new classification method for inpatients with new-onset diabetes in Eastern China. In comparison with previous studies, our study included six other diabetes-related auto-antibodies besides GADA and compared the differences of treatments in China for the first time.

The cluster‐based classification can achieve more refined and balanced diabetes typing. Most of the previous studies observed MARD was the most common subtype (7–11). As for hospitalized adult patients with new-onset diabetes in Beijing, China, Wang et al. found that the proportion of MOD was the highest followed by MARD, SIDD, SIRD, and SAID (12). However, the results of our study differ from previous studies as SIDD was the most common subtype, followed by MARD, MOD, SIRD, and SAID. This may be due to the fact that all of the subjects of our study were hospitalized patients. The blood glucose control of SIDD group was the poorest so that they may prefer hospitalization. Patients in the MARD group had the best blood glucose control, which may reduce the possibility of hospitalization. In the SWAN study, the HOMA-β was lower in Chinese Americans and Japanese Americans when compared with non-Hispanic whites and non-Mexican-American Latinos, suggesting that the β-cell secretion capacity of Asians was lower than that of westerners (28). A study of a comparison of different accelerators to early-onset type 2 diabetes mellitus between Anglo-Celtic and Chinese patients suggested that early β-cell deficiency was an important accelerator for type 2 diabetes mellitus in Chinese population (29). These results suggested that β-cell failure was more significant in Chinese patients than in western patients in the early phase of type 2 diabetes mellitus, further to result in a higher proportion of SIDD patients in the Chinese population than in the Western population. Our study found that GADA was the most common diabetes-related autoantibodies in the SAID group. Previous studies suggested that 70%-80% of newly diagnosed patients with type 1 diabetes mellitus were GADA positive and had a longer duration and higher positive rate of GADA rather than the other antibodies (30, 31). The incidence of type 1 diabetes mellitus varied greatly according to the reports all over the world (32). Western countries, especially Northern Europe, had a high incidence of type 1 diabetes mellitus, while the prevalence among Asian countries including China was low (32, 33). The study also reported that the incidence of type 1 diabetes mellitus in children under 15 years old was positively correlated with latitude, with a higher incidence in Northern China and a lower incidence in Southern China (33). The above three results explained why the incidence of SAID in our study was very low even though we included six other diabetes-related auto-antibodies besides GADA. The clustering characteristics of each subgroup in our study were consistent with the results of Ahlqvist et al. except that SIDD was a late-onset disease. This could be due to the fact that in the study of Ahlqvist et al. patients with other diabetes-related auto-antibodies positive besides GADA might be classified as SIDD, which may lead to patients with SIDD showed lower in age.

HOMA2-β was difficult to reflect the dynamic process of insulin secretion stimulated by glucose. The results of comparisons of C-p levels at five time points and the AUC of 0-180 min C-p levels in our study more strongly confirmed the differences in islet β-cell function among the five subgroups.

The previous studies all found that patients with SIRD were the most likely to develop DKD despite not too bad blood glucose control (7, 9–12). The same was true in our study, but the difference was no longer significant in HOMA2-IR-adjusted risk, further reinforcing the association between insulin resistance and DKD. Patients with MOD and SIRD had a higher levels of TG, blood pressure, and SUA and were prone to get MS. This further proved that insulin resistance and obesity were the core mechanisms of MS. Moreover, Our study found that Patients with SIDD, SIRD, and MARD were more likely to develop CVD and/or stroke, DPVD, and DSPN due to their older age. From what has been discussed above, the SIRD group may be the most serious type among the five subgroups because it was prone to have many serious complications and comorbidities.

Both Wang et al. and Ahlqvist et al. found that DKA was the major complication of SAID and SIDD groups and HbA1c was considered as the strongest predictor (7, 12). We also found that the risk of DKA in SAID group was no longer significantly high after adjusting for HOMA2-β and HbA1c. Both in Nordic and German studies, the SIRD group was more likely to have NAFLD because the TM6SF2 gene usually associated with NAFLD was in SIRD group, but not in MOD group (7, 10). However, our research showed that the highest risk of NAFLD was in the MOD group. The risk was still more than twice as high as the other four groups even after adjusting for HOMA2-IR and BMI, indicating that excluding the effects of BMI and HOMA2-IR, the clustering itself still had a high predictive value for NAFLD. This may be due to the complex pathogenesis of NAFLD, and its intrinsic pathophysiological basis still needed to be further explored.

As for treatments, our study found that as the recognized first-line antihyperglycemic drug, metformin was the most commonly used in all participants. Considering that our study subjects were all inpatients, their blood glucose levels may be higher than those in the whole diabetic population, which leaded to a higher use of insulin in our study. Both Nordic and our studies found that SAID and SIDD groups had the highest usage rate of insulin (7). Patients in these two groups were characterized by poor metabolic control in blood glucose and overt insulin deficiency, so early use of insulin was appropriate. Our study found that α-glycosidase inhibitors were also the most commonly used in patients with SAID and SIDD, because of the higher PBG in these two groups. A study showed that DPP-4 inhibitors with good safety and low incidence of hypoglycemia could be a great choice for elderly type 2 diabete mellitus patients (34). In our study, the usage rate of DPP-4 inhibitors in patients with SIDD, SIRD, and MARD which were characterized by late-onset age was significantly higher than that in patients with SAID and MOD which were characterized by early-onset age. In our study, MOD patients were more likely to use SGLT-2 inhibitors and GLP-1 receptor agonists, which also had weight loss effects. To sum up, according to the results of cluster‐based classification, the current treatments were relatively reasonable. There were researches that showed SGLT-2 inhibitors significantly reduce the risk of major adverse cardiovascular events and related kidney diseases (35, 36). Some studies suggested that GLP-1 receptor agonists can not only reduce weight, but also prevent cardiovascular diseases (37, 38). It reminded us that the initial treatment of SGLT-2 inhibitors may be more effective for patients with SIRD to improve late prognosis. However, in our study, only 24.7% of patients in the SIRD group used SGLT-2 inhibitors, and there was no significant difference in the utilization rate compared with SAID, SIDD, and MARD groups. SIDD and MARD groups with a higher risk of CVD and/or stroke, the utilization rates of SGLT-2 inhibitors and GLP-1 receptor agonists were also about 20%. It showed that although the traditional classification method considered the external characteristics of disease in guiding the formulation of treatment options, it could not identify the potential risk of different patients in the early stage. The cluster‐based classification of diabetes could help to predict the risk of diabetes-related complications and comorbidities and guide treatments, which may make up for the long-term clinical needs that cannot be met by the traditional classification.

Considering that the precise diagnosis and classification of diabetes is still in its infancy, although clustering classification fails to achieve precision in the true sense, it can progress from the original fuzzy typing to relatively precise typing. Clustering classification is a complement to the traditional classification rather than a substitute. The traditional classification is the etiological classification and will continue to be used in the future. These two classifications are not contradictory and can complement each other to better guide treatments.

The cluster‐based classification has been applied to inpatients in National Center of Gerontology, suggesting that the classification system was also applicable to inpatients with diabetes in China (12). This has laid a solid theoretical foundation for our research. However, most of the inpatients in National Center of Gerontology were the elderly, so we further tracked and explored on this basis. Under the three-level medical service system in China, our study included patients from five hospitals at these three levels, so the age of subjects may be more balanced. However, our study also had some limitations. Firstly, Sterling et al. found that patients can move between clusters from baseline to 5-year follow-up (10). However, our study is a cross-sectional study. With the progression of diabetes, the stability of cluster‐based classification and the occurrence or development of diabetes-related diseases were not fully verified. Secondly, differences in response to various treatments among the five subgroups were not explored. Thirdly, GADA and C-peptide assays are expensive, which limits their use in the developing countries. Finally, the small sample size of patients with SAID may lead to be prone to class II errors, so the results of pairwise comparisons between SAID group and the other groups were biased. More prospective studies in larger and more diverse populations are needed to confirm the results of our study in the future. RCTs are also required to assess the clinical utility of any reclassification effort.

## 5 Conclusions

Clustering distribution of inpatients with new-onset diabetes in Eastern China was different to that of participants from other regions and sources. The clustering characteristics of patients in different subgroups were basically consistent with the results of Ahlqvist et al. The clinical characteristics, treatments, and the prevalence and risk of diabetes-related complications and comorbidities of patients in five subtypes were apparently different. Due to late-onset age, severe insulin resistance, and obesity, SIRD was the most severe type and may require early intensive and precise therapies. The current treatment strategies only considered the external characteristics of the patients and had some defects. The new cluster‐based classification might be an important step towards precise treatments for diabetes, which is helpful to realize the personalized managements and treatments of diabetes.

## Data Availability Statement

The raw data supporting the conclusions of this article will be made available by the authors, without undue reservation.

## Ethics Statement

The studies involving human participants were reviewed and approved by The ethics committee of Zhongda hospital (2018ZDSYLL143-P01). Written informed consent to participate in this study was provided by the participants’ legal guardian/next of kin.

## Author Contributions

XS contributed to the conception of the work, data analyses, and drafting the manuscript. Yl revised the manuscript. JS guided the statistical analyses of this study. XS, NH, TY, XW, JZ, ZZ, HG, JL, WZ, HY, QW, and KW made contributions to acquisition of data. LL and KW revised it critically for important intellectual content. All authors approved the final version.

## Funding

This work was supported by National Natural Science Foundation of China (81970717 and 82170845) and Scientific Research Project of Jiangsu Provincial Health Commission (ZD2021007).

## Conflict of Interest

The authors declare that the research was conducted in the absence of any commercial or financial relationships that could be construed as a potential conflict of interest.

## Publisher’s Note

All claims expressed in this article are solely those of the authors and do not necessarily represent those of their affiliated organizations, or those of the publisher, the editors and the reviewers. Any product that may be evaluated in this article, or claim that may be made by its manufacturer, is not guaranteed or endorsed by the publisher.
