# Supplementary material for: Clinical Characteristics of Inpatients With New-Onset Diabetes Mellitus in Eastern China: Based on Novel Clustering Analysis
Source: Front Endocrinol (Lausanne). 2022 Jul 27;13:927661. doi: 10.3389/fendo.2022.927661 (PMC9363570; doi:10.3389/fendo.2022.927661)
Supplement: Supplementary file 1 [file Table_1.docx]

**Supplementary Table 1: Distribution of diabetes-related autoantibodies in SAID group**

| Diabetes-related autoantibodies | No. of patients |
| --- | --- |
| GADA | 24 (57.14%) |
| ICA-120 | 10 (23.81%) |
| ICA-40 | 1 (2.38%) |
| GADA + ICA-120 | 2 (4.76%) |
| ICA-120 + IA-2A | 1 (2.38%) |
| ZnT8A + IA-2A | 2 (4.76%) |
| GADA + ICA-120 + ICA-40 | 1 (2.38%) |
| ICA-120 + ZnT8A + IA-2A | 1 (2.38%) |

GADA = glutamic acid decarboxylase antibody. ICA-120KD = islet cell antibody-120KD. ICA-40KD = islet cell antibody-40KD. ICA-64KD = islet cell antibody-64KD. ZnT8A = zinc transporter 8 antibody. IA-2A = protein tyrosine phosphatase 2 antibody. IAA-5.8KD = insulin autoantibody-5.8KD.

**Supplementary Table 2:** **The risk of diabetes-related diseases among the five subgroups after adjusting related clustering variables**

|  | Variables | OR | 95%CI | | *P* value |
| --- | --- | --- | --- | --- | --- |
|  |  |  | low | high |  |
| DKD | HOMA2‐IR | 1.145 | 1.055 | 1.242 | 0.001 |
|  | SIRD | 1 |  |  |  |
|  | SAID | 0.718 | 0.222 | 2.328 | 0.581 |
|  | SIDD | 0.631 | 0.303 | 1.313 | 0.218 |
|  | MOD | 0.544 | 0.267 | 1.108 | 0.093 |
|  | MARD | 0.675 | 0.305 | 1.497 | 0.334 |
|  |  |  |  |  |  |
| DPVD | Age | 1.061 | 1.046 | 1.077 | < 0.001 |
|  | SIRD | 1 |  |  |  |
|  | SAID | 0.501 | 0.13 | 1.937 | 0.317 |
|  | SIDD | 0.81 | 0.448 | 1.464 | 0.484 |
|  | MOD | 0.866 | 0.4 | 1.874 | 0.715 |
|  | MARD | 0.636 | 0.336 | 1.207 | 0.166 |
|  |  |  |  |  |  |
| DSPN | Age | 1.059 | 1.046 | 1.071 | < 0.001 |
|  | SIRD | 1 |  |  |  |
|  | SAID | 0.937 | 0.389 | 2.257 | 0.884 |
|  | SIDD | 0.963 | 0.574 | 1.613 | 0.885 |
|  | MOD | 1.07 | 0.595 | 1.924 | 0.821 |
|  | MARD | 0.829 | 0.479 | 1.434 | 0.502 |
|  |  |  |  |  |  |
| CVD and/or stroke | Age | 1.106 | 1.09 | 1.123 | <0.001 |
|  | SIRD | 1 |  |  |  |
|  | SAID | 0.915 | 0.318 | 2.634 | 0.869 |
|  | SIDD | 0.681 | 0.379 | 1.223 | 0.198 |
|  | MOD | 0.652 | 0.331 | 1.284 | 0.216 |
|  | MARD | 0.691 | 0.373 | 1.279 | 0.239 |
|  |  |  |  |  |  |
| MS | BMI | 1.522 | 1.424 | 1.627 | < 0.001 |
|  | HOMA2‐IR | 1.289 | 1.166 | 1.425 | < 0.001 |
|  | SIRD | 1 |  |  |  |
|  | SAID | 0.888 | 0.262 | 3.007 | 0.848 |
|  | SIDD | 1.355 | 0.609 | 3.018 | 0.457 |
|  | MOD | 1.165 | 0.51 | 2.662 | 0.716 |
|  | MARD | 2.132 | 0.921 | 4.936 | 0.077 |

DKD = diabetic kidney disease. DPVD = diabetic peripheral vascular disease. DSPN = diabetic distal symmetric polyneuropathy. CVD = cardiovascular disease. MS = metabolic syndrome. SAID = severe autoimmune diabetes. SIDD = severe insulin-deficient diabetes. SIRD = severe insulin-resistant diabetes. MOD = mild obesity-related diabetes. MARD = mild age-related diabetes. OR = **odd ratio. 95% CI = 95% confidence interval.** *P* value < 0.05 was considered significant.

**Supplementary Table 3: The prevalence, una****djusted risk, and adjusted risk of diabetes-related diseases among the five subgroups**

|  |  |  | Model 1 | | | | Model 2 | | | |
| --- | --- | --- | --- | --- | --- | --- | --- | --- | --- | --- |
|  | Variables | Events (%) | OR | 95%CI | | *P* value | OR | 95%CI | | *P* value |
|  |  |  |  | low | high |  |  | low | high |  |
| DR | SAID | 6 (14.3%) | 1 |  |  |  |  |  |  |  |
|  | SIDD | 52 (11.5%) | 0.782 | 0.314 | 1.945 | 0.597 |  |  |  |  |
|  | SIRD | 7 (8.6%) | 0.568 | 0.178 | 1.812 | 0.339 |  |  |  |  |
|  | MOD | 21 (10.1%) | 0.677 | 0.256 | 1.796 | 0.434 |  |  |  |  |
|  | MARD | 14 (5.9%) | 0.378 | 0.137 | 1.048 | 0.062 |  |  |  |  |
|  |  |  |  |  |  |  |  |  |  |  |
| NAFLD | MOD | 190 (91.8%)a | 1 |  |  |  | 1 |  |  |  |
|  | SAID | 16 (38.1%)c | 0.055 | 0.025 | 0.122 | < 0.001 | 0.307 | 0.124 | 0.763 | 0.011 |
|  | SIDD | 243 (53.9%)c | 0.105 | 0.062 | 0.178 | < 0.001 | 0.408 | 0.224 | 0.745 | 0.003 |
|  | SIRD | 58 (71.6%)b | 0.226 | 0.113 | 0.451 | < 0.001 | 0.159 | 0.072 | 0.351 | <0.001 |
|  | MARD | 134 (56.8%)b,c | 0.118 | 0.067 | 0.206 | < 0.001 | 0.431 | 0.231 | 0.804 | 0.008 |
|  | BMI |  |  |  |  |  | 1.212 | 1.154 | 1.274 | <0.001 |
|  | HOMA2-IR |  |  |  |  |  | 1.231 | 1.121 | 1.351 | <0.001 |
|  |  |  |  |  |  |  |  |  |  |  |
| DKA | SAID | 16 (38.1%)^a^ | 1 |  |  |  | 1 |  |  |  |
|  | SIDD | 64 (14.2%)^b^ | 0.269 | 0.137 | 0.529 | < 0.001 | 0.312 | 0.147 | 0.666 | 0.003 |
|  | SIRD | 5 (6.2%)^b,c^ | 0.107 | 0.036 | 0.321 | < 0.001 | 0.9 | 0.216 | 3.743 | 0.885 |
|  | MOD | 38 (18.4%)^b^ | 0.365 | 0.179 | 0.747 | 0.006 | 1.157 | 0.499 | 2.682 | 0.735 |
|  | MARD | 6 (2.5%)^c^ | 0.042 | 0.015 | 0.118 | < 0.001 | 1.054 | 0.311 | 3.576 | 0.933 |
|  | HbA1c |  |  |  |  |  | 1.525 | 1.339 | 1.737 | < 0.001 |
|  | HOMA2-β |  |  |  |  |  | 0.931 | 0.904 | 0.959 | < 0.001 |

DR = diabetic retinopathy. NAFLD = non-alcoholic fatty liver disease. DKA = diabetic ketoacidosis. SAID = severe autoimmune diabetes. SIDD = severe insulin-deficient diabetes. SIRD = severe insulin-resistant diabetes. MOD = mild obesity-related diabetes. MARD = mild age-related diabetes. HbA1c = glycated hemoglobin A1c. BMI = body mass index. HOMA2-β = homeostasis model assessment 2 estimates of β cell function index. HOMA2-IR = homeostasis model assessment 2 estimates of insulin resistance index.

The variables marked with the same letters indicated no significant differencs of pairwise comparisons. The variables marked with different letters were used to indicate pairwise comparisons with significant differences. OR = **odd ratio. 95% CI = 95% confidence interval.** *P* value < 0.05 was considered significant.
